# Tracing anxious lives: a biographical-narrative toolkit for anxieties and coping strategies in three central and eastern European countries

**DOI:** 10.1016/j.mex.2026.104084

**Published:** 2026-08-01

**Authors:** Krzysztof Mączka, Klaudia Rodziejczak, Eszter Markó, Ołena Bodnar-Potopnyk, Oksana Khomiak, Emőke Gondos, Júlia Váradi, Zuzanna Kurowska, Barbara Sikora, Maciej Milewicz

**Affiliations:** a Faculty of Sociology, Adam Mickiewicz University, Szamarzewskiego 89C, Poznan, 60-568, Poland; b Faculty of Psychology and Cognitive Science, Adam Mickiewicz University, Szamarzewskiego 89AB, Poznan, 60-568, Poland; c Department of European Ethnology – Cultural Anthropology, University of Pécs, Rókus street 2, Pécs, 7624, Hungary; d Faculty of Sociology, University of Warsaw, Karowa 18, Warsaw, 00-324, Poland; e Faculty of Humanities, National University of Kyiv-Mohyla Academy, 2 Skovorody Str., Kyiv, 04070, Ukraine

**Keywords:** Biographical interviews, Narratives and narrations, Cognitive interviews, Qualitative content analysis, MAXQDA, Poland, Ukraine, Hungary, Central and Eastern Europe (CEE), Qualitative methods, Anxiety, Coping

## Abstract

Studying anxieties and coping strategies as broad, interrelated phenomena is methodologically demanding, especially in international, multilingual research. These experiences emerge across everyday domains—including health, family, and the environment—while linguistic and contextual differences further complicate comparison.

We address this limitation through biographical-narrative interviews that situate anxieties and coping strategies within individual life courses. The approach elicits detailed accounts of personal histories, with particular attention to environmental anxieties and how participants have navigated them.

The method deliberately situates environmental anxieties alongside other general anxieties reported by participants. This enables researchers to examine environmental anxieties in relation to competing anxieties and may help mitigate single-issue bias. It also generates more context-sensitive findings for policy and practice because environmental anxieties rarely occur in isolation.

This article presents a Biographical-Narrative Toolkit for studying anxieties and coping strategies, developed and applied in comparative research conducted in Poland, Ukraine, and Hungary. The toolkit offers a transferable framework for future studies of anxiety and coping, while also providing methodological inspiration for research beyond this field.

• Multistage codebook development, standardized coder training, paired review, and cross-country code harmonization support consistent code interpretation and qualitative comparability.
• A systematic procedure inspired by quantitative research helps manage interpretation in large, international qualitative research teams.

## Graphical abstract

**Figure:**
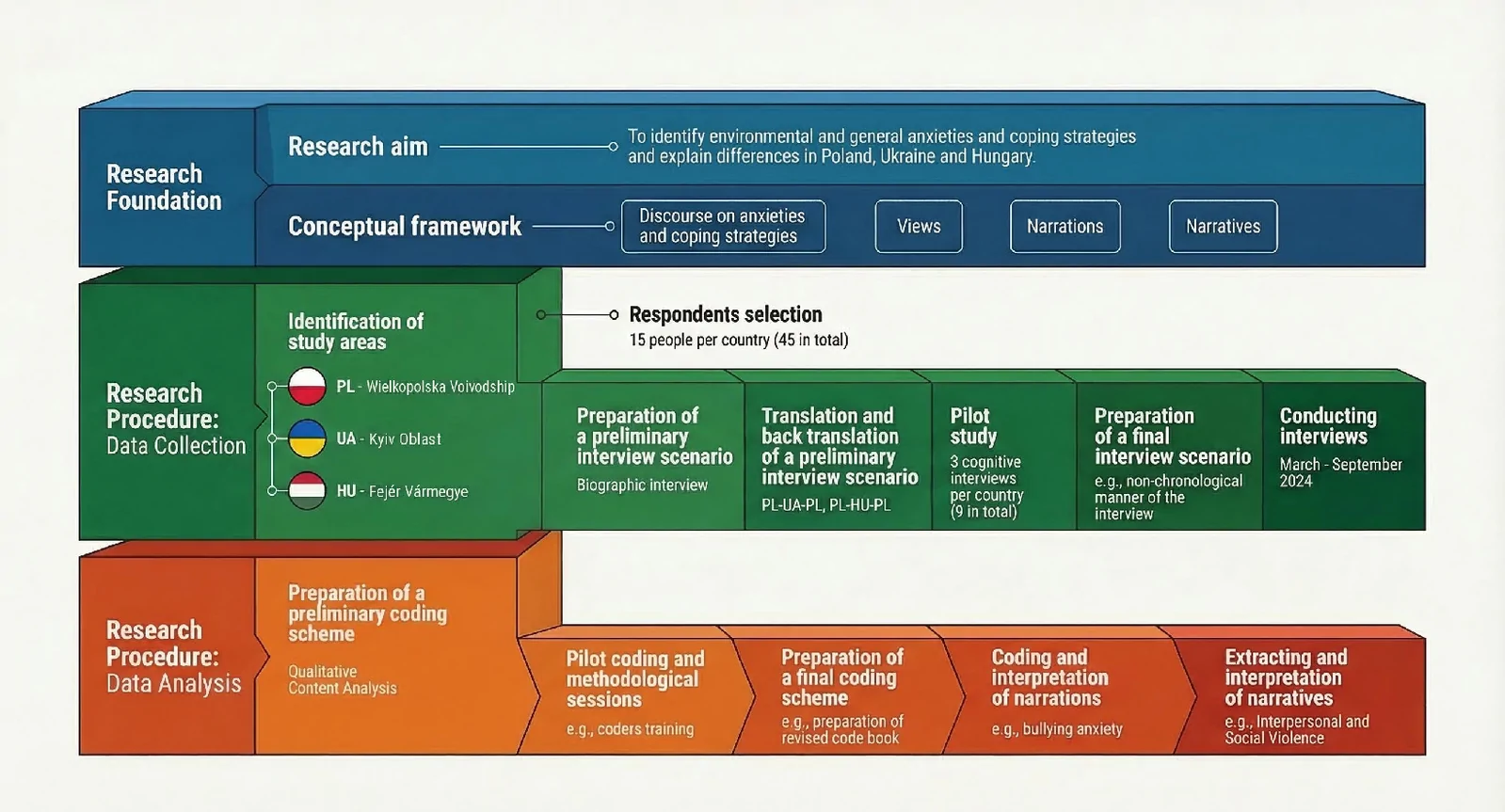

## Specifications table

**Table:** 

Subject area	Psychology
More specific subject area	Sociology, social psychology
Name of your method	A Biographical-Narrative Toolkit for Anxieties and Coping Strategies
Name and reference of original method	Koch, L., Gorris, P., & Pahl-Wostl, C. (2021). Narratives, narrations and social structure in environmental governance. Global Environmental Change, 69, 102,317 [1]. Mayring, P. (2022). Qualitative content analysis: A step-by-step guide [2]. Greene, M. (2018). Paths, projects and careers of domestic practice: Exploring dynamics of demand over biographical time. In Demanding energy: space, time and change (pp. 233–256). Cham: Springer International Publishing [3].
Resource availability	Data: The analysis is based on confidential primary data from biographical interviews. Data may be available upon request. Software: https://www.maxqda.com [4]

## Background

Studying anxieties and coping strategies as broad, multifaceted phenomena is intrinsically challenging. These experiences stem from and affect multiple spheres of everyday life—including physical health, family relations, employment, finances, and the nonhuman environment. In this landscape, knowledge accumulates in fragmented areas, while pressure to publish minimal, incremental "salami-sliced" papers breaks complex lived realities into analytically convenient but substantively thin slices (see, e.g., [5]). The resulting literature is rich in isolated variables and short time horizons but poor in integrative accounts explaining how different anxieties co-occur and elicit distinct or overlapping coping practices. Moreover, the complex lived experiences of individuals facing emotional distress cannot be adequately interpreted unless they are examined through a lens that is equally complex. Therefore, we turn to narrative and acknowledge its key role in understanding coping and anxiety from both psychological and cultural perspectives [6,7].

Recent methodological reviews in environmental research [8,9] argue that qualitative approaches are essential for context-sensitive research that helps explain the complexity of socio-environmental phenomena. They emphasize that narrative, participatory, and other qualitative methods can reveal dimensions of environmental change that remain inaccessible to fragmented and generalizing quantitative designs. At the same time, eco-anxiety research is dominated by quantitative approaches; greater use of qualitative and mixed-method approaches could improve the capture of lived experiences, coping processes, and their evolution over time [10].

Our methodological approach addresses this gap by examining anxieties and coping strategies embedded in the individual life course through biographical-narrative interviews. We build on existing work on biographical interviews, emotional distress, and anxieties to develop a novel methodological combination that elicits a holistic perception of anxieties and coping strategies over the life course. The biographical-narrative component is designed to elicit detailed accounts of individuals' broader histories of anxiety, with a focus on environment-related anxieties and the ways they have navigated them. To complement and discipline interviews, we co-produce visual, descriptive timelines that plot key life events alongside coping strategies (see Section "Description of the final scenario" for details). Together, the proposed approach provides a comprehensive view of anxieties and coping mechanisms as they develop over the long term.

Crucially, while environmental anxieties are the primary focus, participants’ perceptions of other salient anxieties are deliberately incorporated. This design follows the longstanding recommendation that “public concern for the environment should be measured relative to concern over other problems” [11], allowing environmental anxieties to be situated within participants’ broader hierarchies of concern. By enabling participants to discuss different sources of anxiety and compare their relative importance, the approach may help mitigate single-issue bias and provide more context-sensitive insights for policy and practice.

## Method details

### Research procedure

We conducted the research between February 2024 and September 2025 across three countries: Poland, Ukraine, and Hungary. To clarify the research procedure, we first explain the key concepts in the study's conceptual framework. Our conceptual framework for assessing participants' views on anxiety and coping strategies is grounded in narrative theory [1,12]. Narrations and narratives, as parts of discourse, are central to human communication and to our analytical approach. Narrations are verbal descriptions that individuals use to describe their lived experiences—that is, a subjectively constructed reality of "what happened?"—and imagined realities—that is, a subjectively constructed vision of "what would happen if?" Narratives, in turn, are cultural artifacts that are constantly constructed through interactions while simultaneously emerging as cultural products created, transmitted, and transformed through individual narrating activities [1]. In the analysis, narrations were treated as individual, directly coded accounts of anxieties and coping strategies expressed in specific interview fragments. Narratives were identified at a later stage as collective interpretative patterns concerning anxieties and coping strategies, constructed through the comparative synthesis of related narrations across interviews.

### Selection and construction of the research tool

Our study used a qualitative research approach based on biographical interviews conducted in three countries. We selected biographical interviews because they enable the collection of data that 1) provide material for an in-depth analysis of anxieties and coping strategies throughout participants' life histories and 2) facilitate a better understanding of individual experiences by providing broader context.

Based on a review of the literature and the research questions, we prepared a preliminary version of the biographical interview scenario and discussed it at a seminar organized by the Qualitative and Mixed Methods in Clinical Psychology Research Lab at the Faculty of Psychology and Cognitive Science, Adam Mickiewicz University in Poznań, Poland. We then created the full version of the scenario and discussed it again at the same institution.

To test the scenario, we conducted an internal pilot study and interviewed two researchers from our team. Both interviews were conducted in Polish, lasted approximately 2 h, and were held online using Microsoft Teams, with both participants' cameras turned on. We used Google Jamboard as an auxiliary tool for creating a timeline. After the interviews, we jointly evaluated the research tool.

The tool was evaluated positively: the scenario elicited in-depth answers to the research questions and did not evoke negative feelings during or after the interviews among the researchers who served as participants. The participants emphasized the positive feelings associated with the opportunity to organize and comprehensively review their life experiences in the context of anxieties and coping strategies. From the interviewers' perspective, the scenario was clear and allowed an in-depth exploration of the required topics. Nevertheless, the internal pilot study helped us better understand the participants' situation, refine the instructions for interviewers, improve the wording of the questions, and prepare an online backup option for data collection. This option was considered especially important for the Ukrainian case because of the Russian invasion. Given the overall positive assessment, we proceeded to the next stage: translating the scenario.

### Translation and back-translation of the scenario

We created the original scenario in Polish and then had professional translation companies translate it into Ukrainian and Hungarian. Next, we used the back-translation method [13] to ensure consistency of meaning across languages. Each version of the research tool was translated back into the source language (Polish) and compared with the original to identify inconsistencies. In the Ukrainian case, back-translation confirmed the high quality of the initial translation and that all questions were consistent with the original Polish version. In the Hungarian case, significant grammatical and syntactic changes were needed to ensure that the content was equivalent in meaning and formulated in accordance with Hungarian oral and written standards. In addition, given the qualitative nature of the research, having native speakers of all the respective languages on the analysis team proved essential during later stages. This arrangement allowed potential inconsistencies arising from misinterpretation or a lack of equivalence in the lexical semantics of the languages to be identified, assessed, and discussed.

### Pilot study

To test the translated versions of the scenario with the target group, we conducted three pilot cognitive interviews in each country—a total of nine interviews—between February and March 2024. Cognitive interviewing is designed to explore the cognitive processes through which participants interpret and answer questions [14,15]. In this context, its primary objective was to ensure before fieldwork that participants' understanding of key questions aligned with the researchers' intentions. Cognitive interviewing techniques enable researchers to identify potential problems with specific questions in the research tool and understand the reasons underlying those problems. They also provide an opportunity to improve questions and reduce participant burden. Specifically, cognitive interviewing can inform researchers about:

1. How participants perceive and interpret questions and expressions and whether they perceive the questions as the researchers intended;
2. What various words and expressions mean in different contexts in light of social, environmental, and cultural norms;
3. Content or structural features of a questionnaire that may cause misinterpretation or refusal to answer.

The cognitive interview format allowed us to evaluate the scenario thoroughly in practice by obtaining answers to the relevant questions in the interview script and observing the cognitive processes that participants used when answering and analyzing the questions. We used three techniques during the cognitive interviews: 1) the think-aloud technique, in which participants answered each question while reporting their related thought process; 2) the probing technique, in which the researcher asked direct supplementary questions; and 3) observation of participants' behaviors. The full cognitive interview scenario is available in Supplementary Materials 1.

The pilot interviews were conducted in a mixed format: six were held in person at participants' or researchers' residences, a university office, or public places (cafés), and the remaining three were conducted remotely via MS Teams and Google Jamboard. We used a mixed-format pilot study to assess how the interview procedure performed under different conditions. This approach was particularly relevant for two reasons: 1) it prepared us to continue research with residents of Ukraine online if war-related restrictions prevented in-person meetings, and 2) it provided an alternative when participants could not provide adequate space in their homes. Google Jamboard became unavailable in early October 2024, but numerous alternatives are available for future use [16], such as Canva's Free Online Whiteboard [17]. The pilot study involved people aged 24–50 with diverse educational backgrounds and places of residence (urban/rural) (Table 1). The pilot interviews lasted 72–220 min.

**Table 1:** Pilot study sample description.

No	Country	Participant ID^1^	Place of residence (urban/rural)	Gender	Age	Education	Interview form (in-person/online)
1	Poland	PL_K_27_63	Urban	Female	27	Secondary	In-person
2	Poland	PL_M_25_210	Urban	Male	25	Secondary	In-person
3	Poland	PL_K_50_220	Rural	Female	50	Higher	Online
4	Ukraine	UA_K_34_150	Urban	Female	34	Higher	In-person
5	Ukraine	UA_M_24_120	Urban	Male	24	Secondary	Online
6	Ukraine	UA_M_39_180	Rural	Male	39	Higher	In-person
7	Hungary	HUN_F_25_83	Urban	Female	25	Higher	In-person
8	Hungary	HUN_M_33_131	Urban	Male	33	Higher	Online
9	Hungary	HUN_F_28_72	Urban	Female	28	Higher	Online

Source: the authors.

1 Participant ID structure: country initials_gender of the participant_age of participant in years_time of the interview in minutes.

After the pilot study, we amended the scenario's content and structure. For each country, we prepared a report on the cognitive interviews with recommendations for changes (Picture 1 and Supplementary Materials 2).

**Picture 1:**
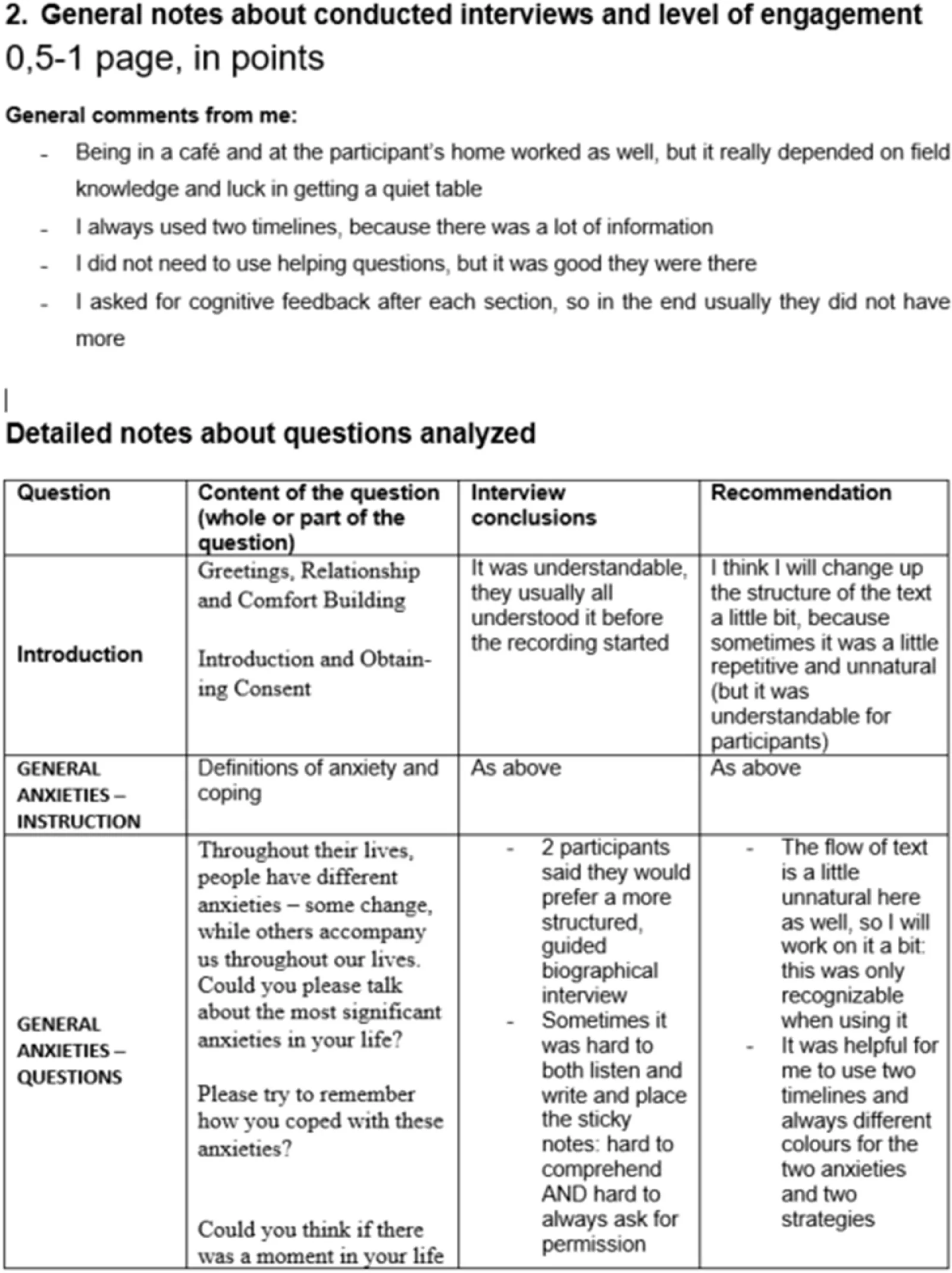
Excerpts from the report on the cognitive pilot interviews in Hungary.

Source: the authors

We discussed all observations and proposed changes during a methodological meeting and agreed on the revisions. Key changes included:

(1) Allowing participants, through the instructions, to tell stories about anxieties in a nonchronological manner;
(2) Expanding the question about anxieties related to the natural environment so that the instructions covered both anxieties about climate change and anxieties about other natural phenomena;
(3) Adding supplementary questions to the second part of the scenario to explore environmental anxieties in greater depth;
(4) Separating the questions about the most important anxieties and the most important coping strategies in the third part of the scenario.

### Description of the final scenario

The final scenario consisted of five parts (Supplementary Materials 3). The first part included an introduction and an outline of the interview, ensuring that participants were thoroughly familiar with the interview process. During this part, the researchers built trust and fostered participants' sense of comfort.

The second part of the scenario addressed the participants' most significant anxieties in life and the strategies they used to cope with them. The researchers presented definitions of anxiety and coping:

**"Anxiety** means feeling worse because you anticipate that something bad might happen and that you will not be able to cope with it."

**"Coping** means actions or ways of thinking used in a situation when you experience anxiety, aiming to improve well-being and reduce feelings of anxiety."

After presenting the definitions, the researcher provided the following context: "People have different anxieties throughout their lives—some change; others stay with us throughout our whole lives." The researcher then asked the leading question: "Can you tell me about the most important anxieties in your life?" If necessary, we asked additional questions.

The third part of the scenario focused on environmental anxieties and ways of coping with them. The order of the questions—first about general anxieties and then about environmental anxieties—was purposeful. It prevented the researchers from suggesting that all anxieties had to be related to the natural environment. However, if environmental anxieties were relevant to participants, they might have raised the topic independently in the first part of the interview, which focused on the most important anxieties. The researchers asked the following question: "As we have already said, people have various anxieties during their lives, sometimes also related to the natural environment. Please tell us if you experience or have experienced such anxieties."

In the fourth part of the scenario, the researchers asked participants to indicate which of the listed anxieties and coping strategies had the most significant impact on their lives and to justify their choices. We also asked participants which set of anxieties—general or environmental—was more important to them.

The fifth part of the scenario involved winding down the interview. The researchers asked about participants' feelings and reflections after the conversation and inquired about their well-being. The recording was then stopped, participants received a voucher and a gift, and the conversation shifted to another, preferably neutral topic, concluding the meeting.

We used a life timeline as a supporting interview tool. The timeline was intended to minimize the influence of current events on the interview content and facilitate a focus on participants' memories across their lifespans. It was presented as a black arrow on a white A3 sheet of paper titled "The Timeline of Your Life." During the conversation, the researchers noted in chronological order the anxieties and coping strategies identified by the participant. They recorded anxieties and coping strategies on colored sticky notes, with different colors representing general anxiety, environmental anxiety, coping strategies related to general anxiety, and coping strategies related to environmental anxiety. This tool was intended to support the creation of narrations, connect coping strategies with specific anxieties, and facilitate the identification of the most important anxieties and coping strategies in the fourth part of the scenario.

### Research sample

The selection of Poland, Hungary, and Ukraine resulted from the comparative design of the broader research project, in which several complementary methods were applied in the same three countries. The countries share broadly comparable environmental conditions and a post-communist historical legacy but differ in their exposure to the war in Ukraine and in levels of power distance, which may influence the strategies individuals use to cope with anxiety. According to Hofstede’s index [18], power distance is highest in Ukraine (92/100), followed by Poland (68/100) and Hungary (46/100).

For the biographical study discussed in this article, we conducted interviews in Wielkopolska Voivodship in Poland, Kyiv Oblast in Ukraine, and Fejér Vármegye in Hungary. The regions were selected according to three criteria: 1) they should not be border regions or 2) capital regions, nor 3) should they differ markedly from the national context in socioeconomic structure, population composition, wealth, tourism, or distinctive geographical features such as mountains or coastline. These criteria were intended to limit the influence of region-specific conditions and identify relatively typical national settings. In Ukraine, however, security and organizational constraints related to the ongoing war required a compromise. Kyiv Oblast borders Belarus and includes the national capital; therefore, participants were recruited primarily from its southern part, several hundred kilometers from the border, and only two participants lived in the city of Kyiv.

The research sample was diverse in age, gender, education, and place of residence (rural or urban). Fifteen interviews were conducted in each country, for a total of 45. Previous qualitative interview studies indicate that a sample of approximately 15 participants is generally sufficient to capture a meaningful range of perspectives while allowing in-depth analysis [3]. We therefore adopted 15 interviews per country as the initial target. After a preliminary review of the collected material, we decided not to expand the sample because the final interviews did not yield substantively new content. On this basis, we considered theoretical saturation satisfactory [19]. Table 2 presents the detailed sample structure.

**Table 2:** Research sample description – final study.

Variable	Poland (N = 15)	Hungary (N = 15)	Ukraine (N = 15)	Total (N = 45)
Age (years)				
M; SD	M = 47.07; SD = 16.46	M = 46.4; SD = 15.78	M = 46.73 SD = 16.09	M = 46.73 SD = 15.74
Range	22–81	18–73	18–69	18–81
Gender				
Women	7	7	8	22
Men	8	8	7	23
Education				
Primary or vocational	3	4	1	8
Secondary	7	8	7	21
Higher	5	3	7	15
Residence				
Urban	8	8	8	24
Rural	7	7	7	21

Source: the authors.

### Data collection

The interviews were conducted between March and September 2024 by six trained social researchers (three from Poland, two from Ukraine, and one from Hungary). Initially, an external company recruited participants and provided potential participants with brief information about the topic and study conditions. The researchers then contacted the recruited participants by phone to conduct screening interviews, each lasting about 15 min. These interviews familiarized participants with the study's procedures and objectives in detail. The researchers also assessed participants' willingness to participate in a longer biographical interview and their comfort discussing personal, emotionally difficult topics. If participants confirmed their willingness to participate, the meeting place and date were set during the call. The basic requirements for the study location were 1) a comfortable space where the researcher and participant could sit freely, 2) no third parties in the room, and 3) a surface, preferably a table, on which an A3 sheet of paper with a timeline could be spread out. The researcher first asked whether the participant's residence met these requirements. If not, an alternative interview location was agreed upon. When scheduling interviews, we also ensured that appointments did not follow a long or difficult workday or other stressful activities.

All interviews were conducted in person without online tools. Most interviews (PL: 14, UA: 6, HU: 12) were conducted at participants' residences, while the remainder took place elsewhere: 1) in a university office (PL: 1), 2) in a café (UA: 5, HU: 2), or 3) at a participant's workplace (UA: 4, HU: 1). The interviews lasted 20–241 min (approximately 120 min on average) and were recorded. No minimum duration was set. An interview was accepted for analysis if 1) the participant answered all key questions and 2) the participant described their anxieties and coping strategies within a biographical context. Including interviews of varying lengths preserved diversity in how participants discussed their anxieties and coping strategies rather than favoring only those with highly elaborate life stories. After the interview, participants received a gift voucher worth approximately USD 70, a ceramic mug with the university logo, and a tote bag. The researchers then completed a report on the study procedure (Supplementary Materials 4) and provided the interview recording and photographs of the timeline (Picture 2). The recordings were subsequently transcribed verbatim.

**Picture 2:**
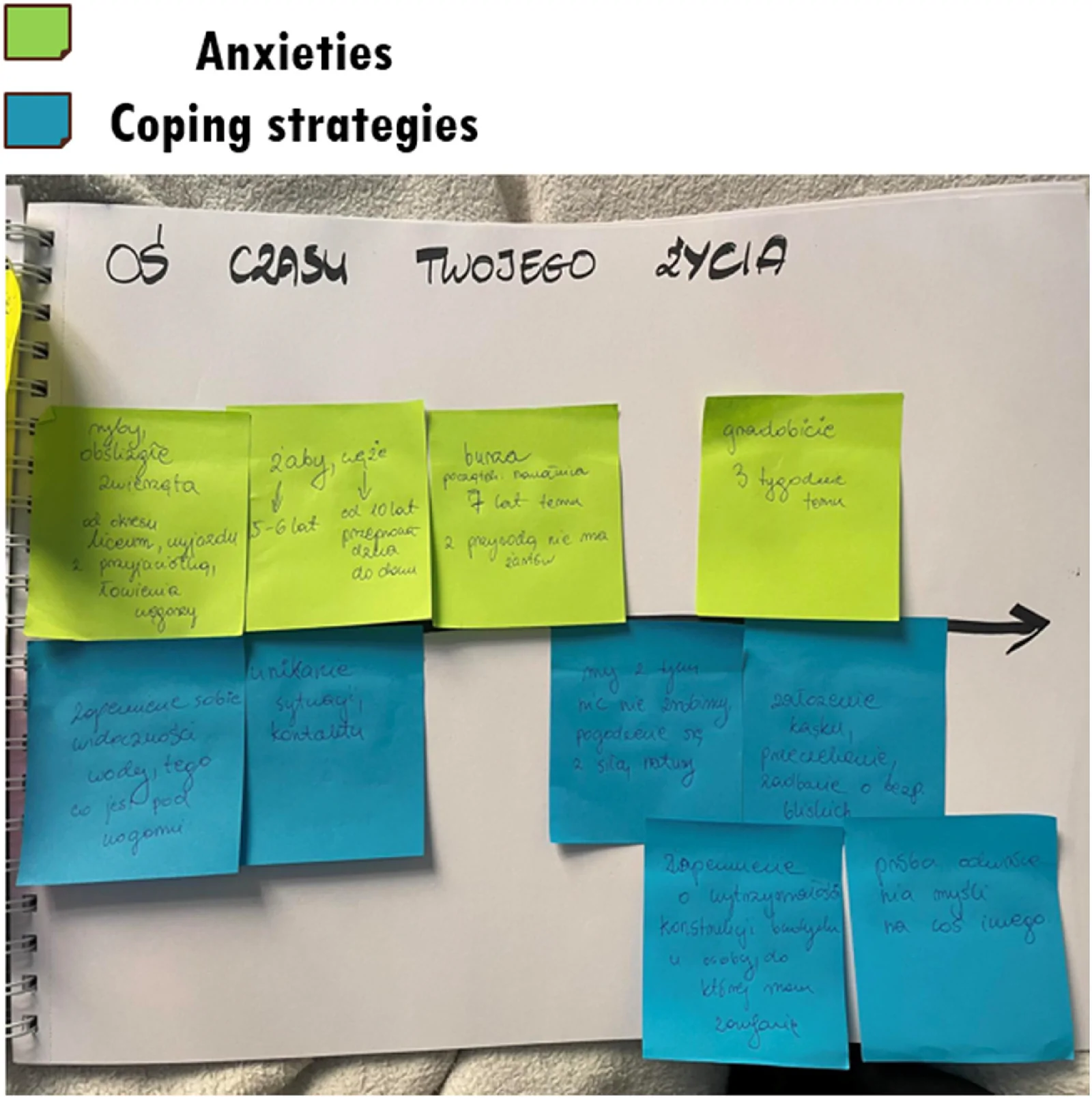
An example of "the timeline of your life" from a polish interview.

Source: the authors

### Qualitative analysis

We used Philipp Mayring's qualitative content analysis (QCA) [2] to analyze the interviews. Data analysis took place between October 2024 and September 2025. QCA combines advantages of a quantitative approach, such as a controlled procedure and strictly defined analytical steps, with a qualitative paradigm. In addition, QCA's deductive-inductive approach allows researchers to use categories derived from the theoretical framework and create new categories based on empirical data. This approach was particularly relevant to the present study because it involved extensive and diverse research material in an international context. Beginning with a deductive framework of code categories and introducing new codes based on interview content organized the material and facilitated an in-depth interpretation. This structured and transparent analytical approach supported consistent coding across a large research team and comparisons of the data across country cases.

#### Coders

The interviews were analyzed by nine coders: four in the Polish subteam, two in the Ukrainian subteam, and three in the Hungarian subteam. Eight coders were women and one was a man. All researchers had social science backgrounds and worked in sociology (three researchers), psychology (two), cultural and social anthropology (three), or history (one). All had prior experience in qualitative data analysis. Four of the nine coders also conducted interviews.

#### Preparation of the code tree, codebook, and pilot coding procedure

First, based on the research questions, interview scenario, and theoretical models, we prepared a code tree. It served as the basis for organizing the material during analysis and as a preliminary framework that coders could develop inductively from the interview content. The code tree was divided into the main code categories, including general anxiety, environmental anxieties, and coping strategies (emotion-focused, problem-focused, and meaning-focused). At this stage, we also prepared the first version of the codebook, which provided detailed coding instructions. Consistent with the distinction between narrations and narratives, the code tree and codebook were used to organize the coding of individual narrations while remaining open to inductive codes emerging from the interview material. Collective narratives were not predefined in the code tree but were constructed during the subsequent post-coding analysis (see Section "Post-coding analysis and comparative analysis" for details).

To account for inductive codes emerging from the interviews, we conducted pilot coding of nine interviews (three per country). These interviews involved participants of different genders and ages to capture the broadest possible range of perspectives. We held meetings with the country subteams to introduce coders to the QCA method as adapted to the study's needs and to the pilot coding procedure. Coders received coding instructions and a deductively developed code tree. They then conducted the inductive analysis in pairs using MAXQDA 24.9.0. As outputs of the pilot coding, the coders submitted nine coded interviews and coding reports. The reports included their reflections and questions about difficulties related to the analyzed material and the coding process. The codebook and coding report were prepared as shared online files, facilitating joint reflection, coordination, and agreement on instructions and codes.

After completing the pilot coding procedure, two researchers—the coordinator of biographical research and the principal investigator—held seven working meetings totaling approximately 40 h. During these meetings, they synthesized the codes, developed a second version of the code tree, and updated the codebook. In addition, two Polish-speaking team members reviewed the code tree and coding instructions. One was involved in the coding process, whereas the other was not, providing different perspectives on the material. This work produced the final versions of the code tree and codebook. The most important changes included:

1. Examples of coded fragments in the codebook were corrected to reflect the meanings of the codes accurately;
2. The inclusion and exclusion criteria for coding quotations as particular narrations were corrected;
3. Some coping strategies that the coders considered overly detailed and specific were synthesized.

#### Coders' training and post-training amendments

An eight-page excerpt from the transcript of a Polish interview (M_56_PL_145) was prepared as coder training material. The interview was suitable for training because it addressed various general and environmental anxieties and included a story spanning several stages of the interviewee's life. The principal investigator translated the transcript into English using DeepL [20] and Grammarly [21].

For training purposes and the final analysis, the coders received 1) a MAXQDA file containing a code tree (Picture 3); 2) a codebook that included frequently asked questions and necessary instructions, a guide to the coping strategy model based on Lazarus and Folkman's theory [22], and a coding report template (Picture 4, Picture 5, Picture 6 and Supplementary Materials 5); and 3) an instructional video summarizing the coding process (Supplementary Materials 6). The codebook was prepared as a shared online file (Picture 5).

**Picture 3:**
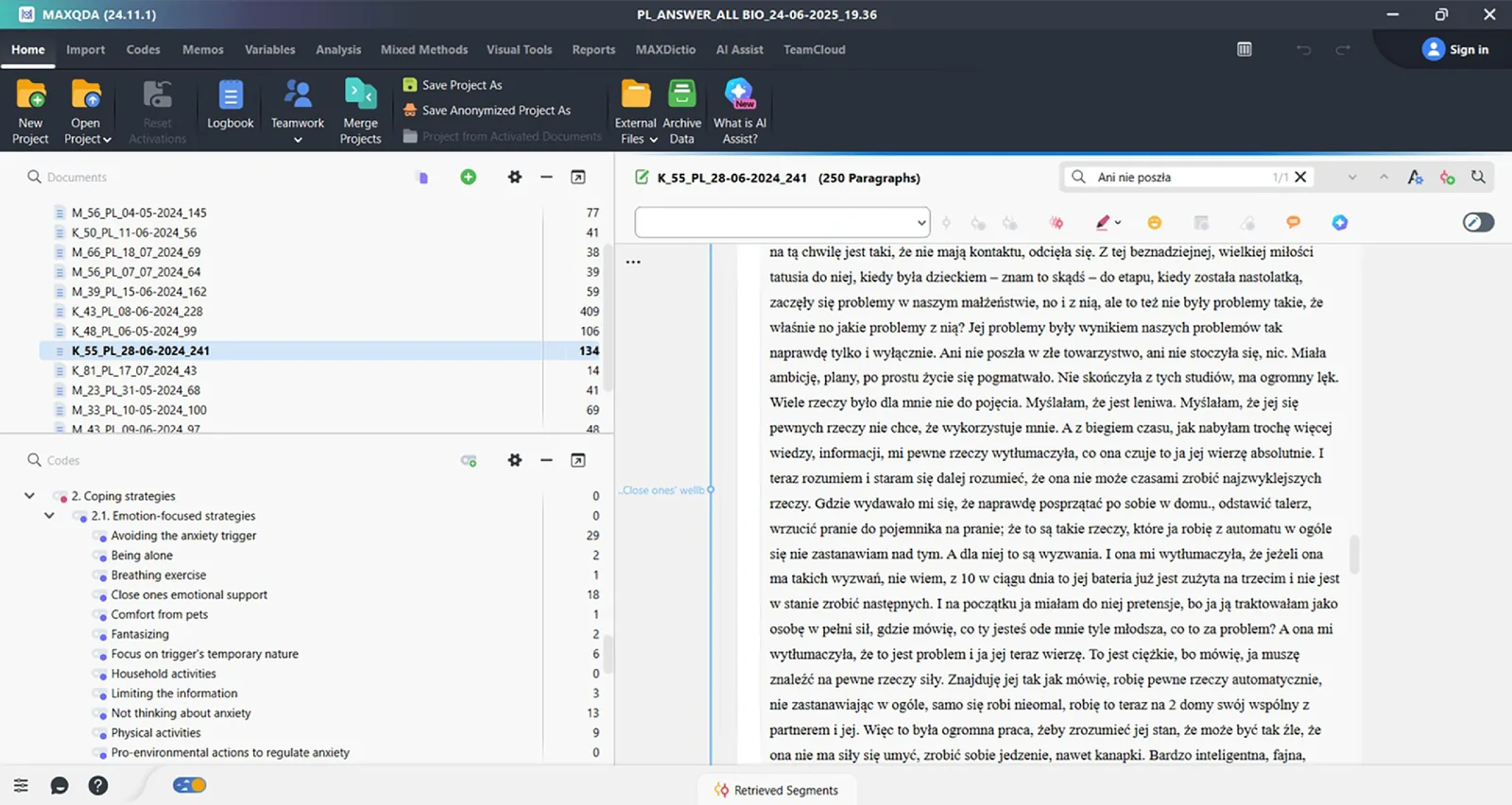
Excerpt from a MAXQDA file showing fragments of the code tree, a transcription, and the list of transcriptions.

Source: the authors

**Picture 4:**
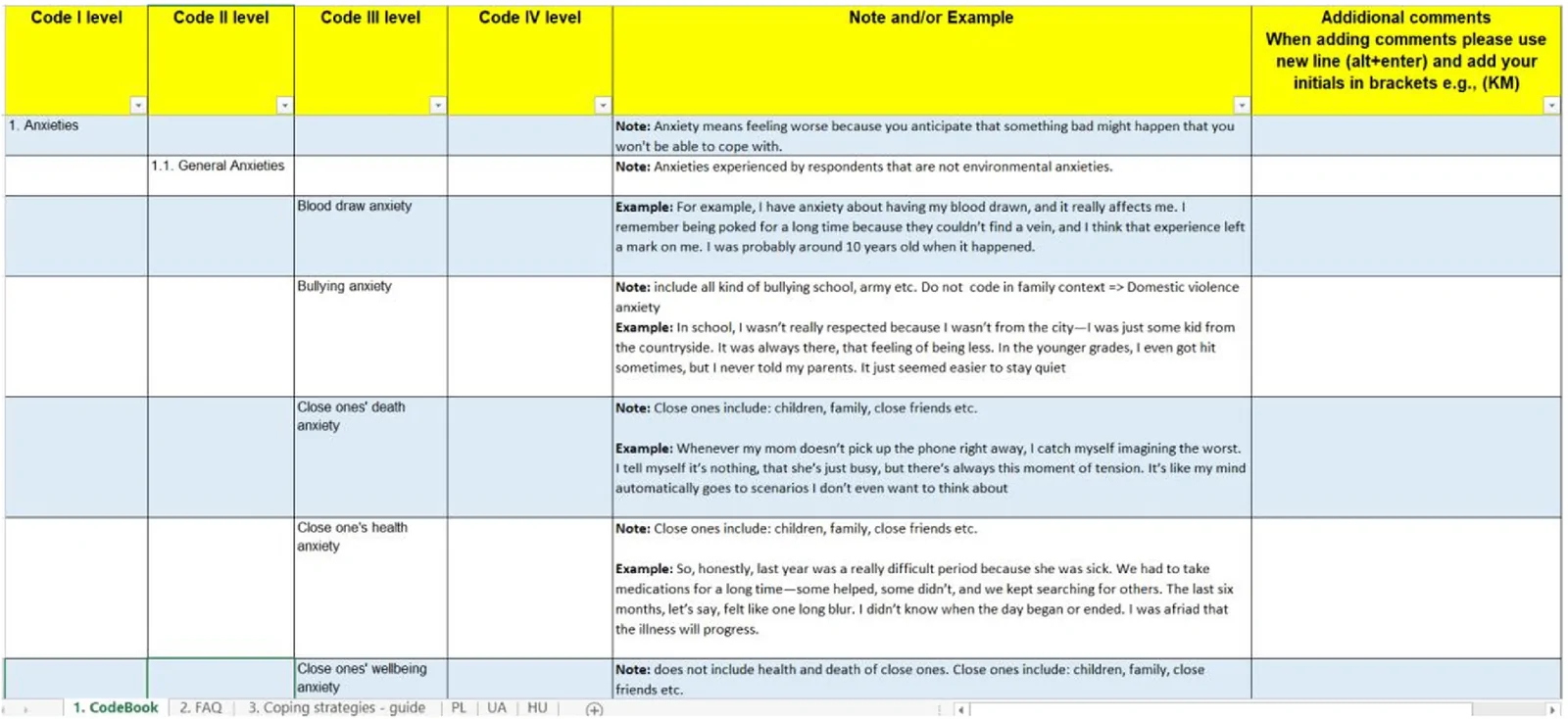
Excerpt from the codebook.

Source: the authors

**Picture 5:**
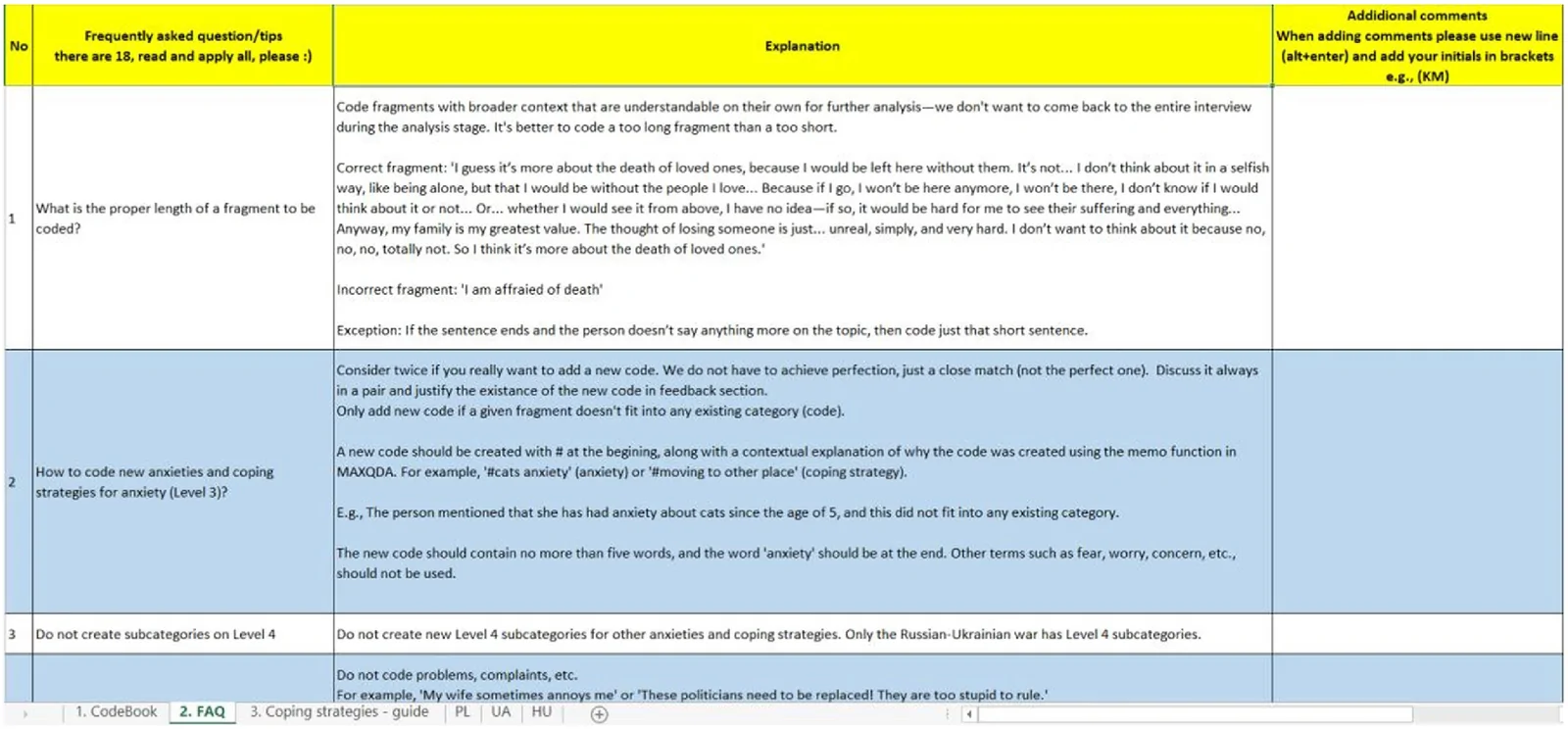
Excerpt from frequently asked questions.

Source: the authors

**Picture 6:**
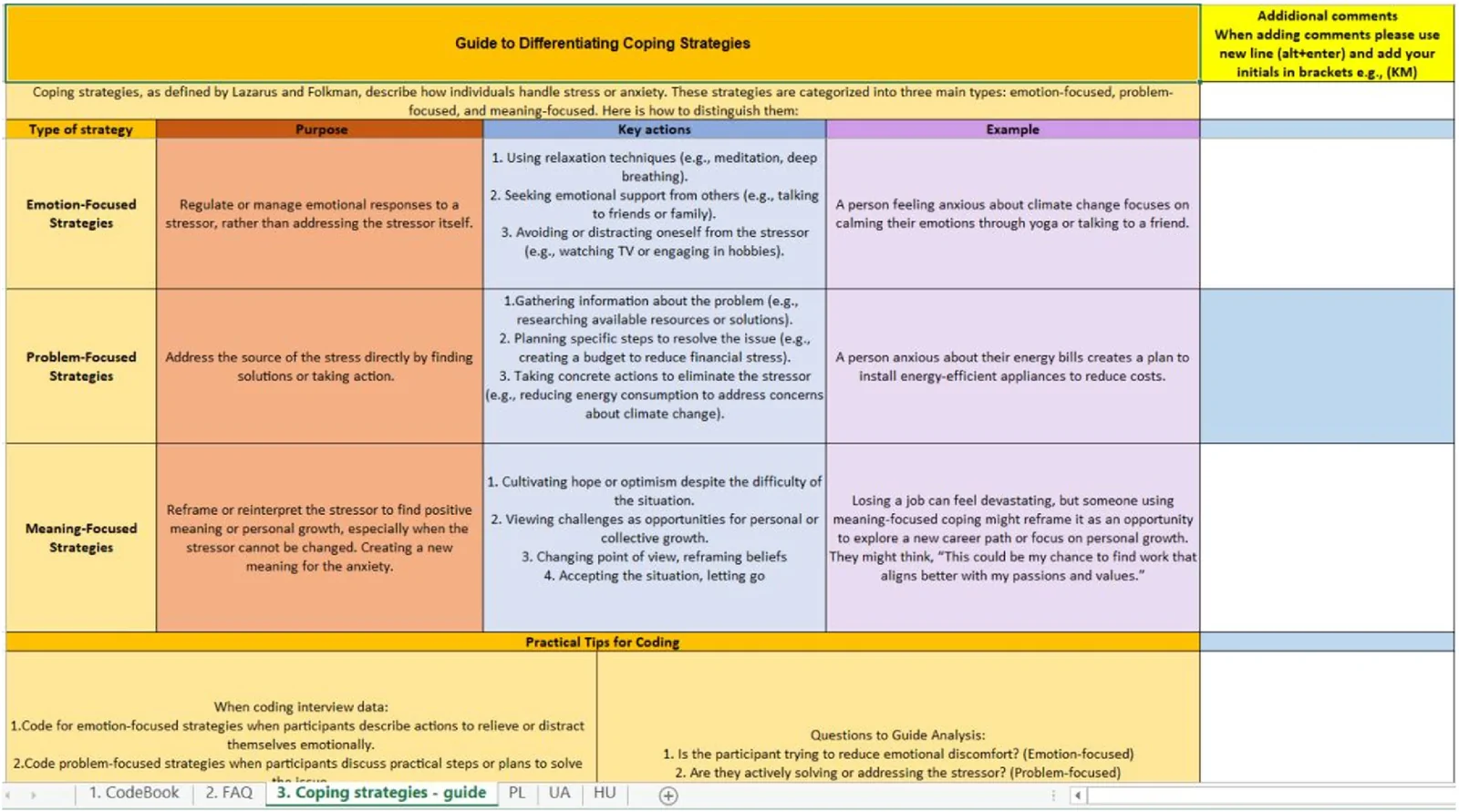
Excerpt from coping strategies guide.

Source: the authors

The coders' tasks were to 1) familiarize themselves with the instructional video and codebook and note any questions about the codes or instructions; 2) code the interview excerpt individually during the training; and 3) prepare a report on the coding process that included general reflections, difficulties and questions encountered, and suggestions for improvement.

All coders participated in the training coding process except the coordinator of biographical research and the principal investigator, who had been most involved in creating the code tree and analyzing the coders' work. These researchers had also coded the training material during the pilot coding used to create the code tree.

After the training coding, the coordinator of biographical research and the principal investigator held two working meetings (10 h in total). They compared the coding of all coders to assess consistency and identify discrepancies. For this purpose, they used the MAXQDA function Analysis => Summary Grid, which enabled them to identify differences in the codes applied by the researchers and compare the content of coded segments. We examined instances in which some researchers coded a segment and others did not, as well as instances in which the same segment was assigned different codes. The team discussed these discrepancies to develop a shared understanding of the codes. This approach followed the qualitative inter-coder consistency guideline proposed by Cofie et al. [23], which emphasizes dialogue and a shared understanding of codes as necessary for consistency in qualitative analysis. In addition, we analyzed the coders' questions and concerns and revised the coding procedure and codebook accordingly. The most important changes were 1) postponing the analysis of changes in the dynamics and presence of individual anxieties and coping strategies throughout participants' biographies until a later stage of the project and 2) synthesizing some coping strategies—particularly by reducing the number of codes in the "problem-focused strategies" category—to maintain the clarity of the code tree and reduce errors caused by too many codes.

After these revisions, we held a meeting with all coders to discuss the analyzed material and resolve any remaining questions. We then made the final changes to the codebook and code tree.

The final codebook and code tree contained three levels of codes representing individual narrations. The first two levels resulted from deductive analysis and the organization of code categories; the third contained inductively generated codes based on interview content (Picture 4 and Supplementary Materials 5). Within this structure, the main narrations included codes related to anxiety, divided into general life anxieties and environmental anxieties. Content was also coded according to coping strategies, which were divided into emotion-focused, problem-focused, and meaning-focused strategies. Additional narrations were coded according to the importance of general life and environmental anxieties and the most important anxieties and coping strategies in participants' lives. Operationally, coders assigned narration codes to specific interview fragments. For example, a participant's account of anxiety that drought could threaten their livelihood was coded as an individual narration concerning drought anxiety.

#### Coding process and validation

As in the pilot coding, the coding procedure was conducted in country subteams, which analyzed material in the original language. Because of the composition of the research team, the Polish and Ukrainian subteams analyzed interviews in fixed pairs, whereas the Hungarian subteam used alternating pairs. The coders used the code tree and codebook developed through the pilot coding and training. Interviews were assigned so that each pair received similar amounts of material to code and review, with an even distribution across gender and age groups. Coding in pairs served as a validation method and followed this procedure. First, the coder read the interview and coded the content in MAXQDA. In cases of doubt, the coder added a note (memo) to the text or code tree and could use the "doubtful" code category within each main code category. The coder then sent the coded file to the other member of the pair, who served as the reviewer. The reviewer read the interview, examined the coded fragments and the coder's questions, and, when needed, identified fragments that should be coded differently.

Next, the coder and reviewer met to discuss any questionable aspects. If interpretative questions remained, they contacted the coordinator and discussed the relevant interview segment with the rest of the team. Because the main analysis involved a review of the coding and joint decision-making rather than two independent codings, a quantitative assessment of independent intercoder agreement was not applicable at this stage. New codes could be created only when an interview fragment could not be interpreted within the existing codes. When proposing a new code, coders were therefore required to include its name and a justification in the report (Picture 7). After completing the procedure, the coder uploaded the interview file to a cloud drive, and the reviewer completed the coding process report. Each pair alternated the coder and reviewer roles across interviews.

**Picture 7:**
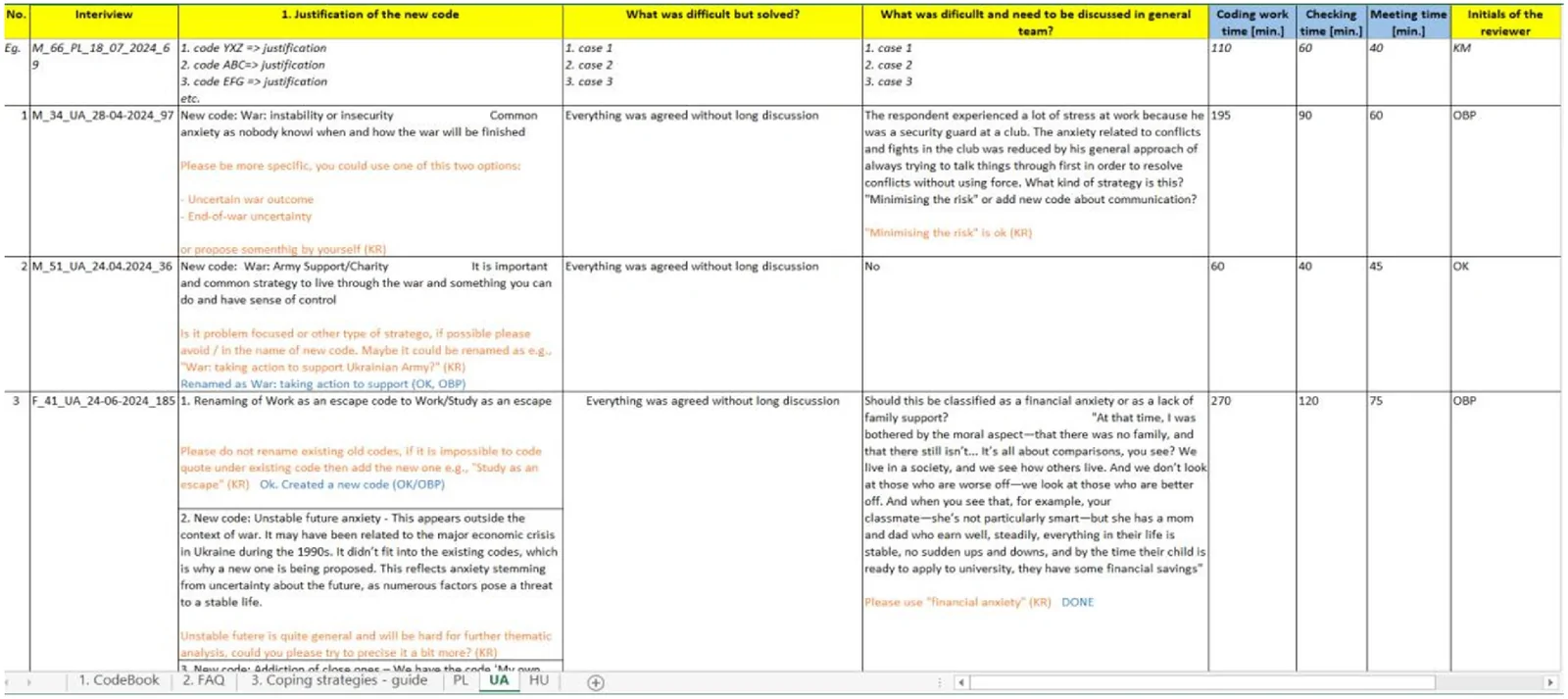
Excerpt from the coding–reviewer table for Ukraine.

Source: the authors

Together, these procedures constituted a systematic cross-country code harmonization process. Harmonization was supported by pilot coding in all three countries; iterative revision of the code tree and codebook; standardized coder training; comparative review of training outputs; paired coding and validation; team discussions of discrepancies; and post-coding standardization of inductively generated codes. Although formal inter-coder agreement coefficients were not calculated, these procedures were designed to promote a shared understanding and consistent application of codes across country teams, thereby supporting qualitative comparability.

#### Post-coding analysis and comparative analysis

After completing the coding process, two researchers—the coordinator of biographical research and the principal investigator—analyzed the coded material. First, we held working meetings to assess whether the newly created codes could be synthesized across interviews from different countries. This procedure was necessary because each interview had been coded independently, so some codes were repeated across interviews. Codes with identical meanings were combined, and their names were standardized. For example, we created the code "animal anxiety" to encompass all codes related to anxiety about particular animal species (e.g., mice, spiders, and ticks). Because this synthesis was conducted after the completion of the main coding phase, merged codes were reflected in the final analytical results rather than retrospectively incorporated into the codebook used during coding process.

Next, narrations were grouped into categories, understood as sets of thematically related content, enabling the identification of collective narratives within each country case (Picture 8). For example, individual narrations coded as "drought anxiety" and "flood anxiety" were grouped into the collective narrative “natural disasters.” This narrative was interpreted as reflecting anxiety arising from limited personal control over natural hazards and their potential consequences, such as disruptions to food production and everyday security.

**Picture 8:**
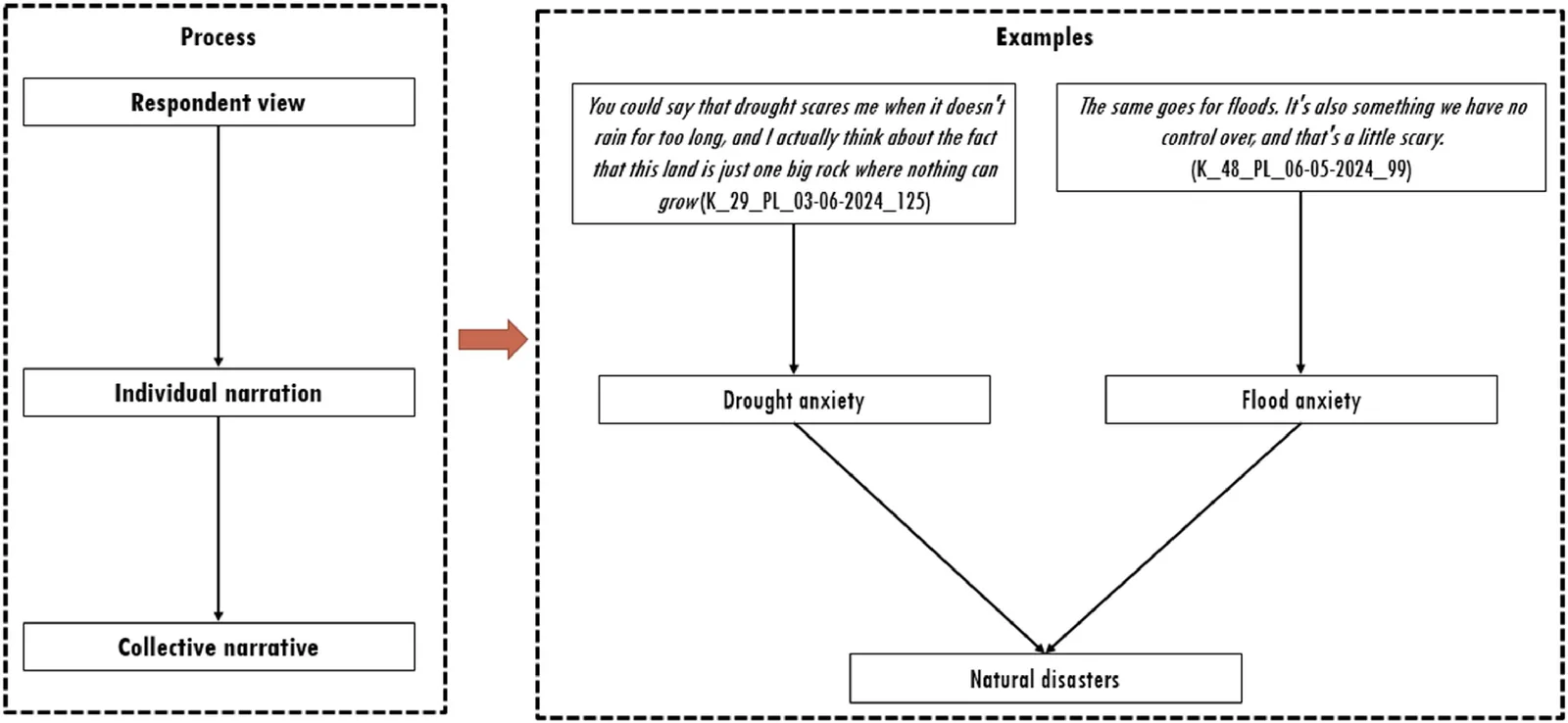
Logic of the interpretation process.

Source: the authors

Thus, narrations represented directly coded accounts of individual participants’ views, whereas narratives were collective interpretative patterns developed by synthesizing related narrations. The results were presented to and discussed within country subteams of the research team. Based on these discussions, we reassigned some narrations to specific narratives and renamed others to better reflect their content.

The frequencies of code application across countries (Supplementary materials 5) for particular narrations show that the interview design captured both environmental and general-life anxieties. General-life anxieties received more coded references and were more often identified by participants as more important in the final comparison. These descriptive indicators suggest that environmental concerns were situated within participants’ wider hierarchies of anxiety rather than examined in isolation. However, because the study did not include a comparison with a single-issue structured interview, the findings demonstrate the breadth of the collected material but do not directly establish the extent to which single-issue bias was reduced.

## Policy and practice implications

Our Biographical-Narrative Toolkit for Anxieties and Coping Strategies offers a detailed and replicable framework for conducting qualitative research. It covers the formulation of the research problem, development and piloting of the interview guide, translation into national languages, coding procedures, and codebook management. By presenting the entire research process rather than only its final analytical stage, the toolkit may support both research practice and the methodological education of students. It can be applied in other Central and Eastern European countries and, with contextual adaptation—particularly of the codebook and code tree—in other regions of the world.

The narratives identified using the toolkit—particularly those concerning security-related and environmental anxieties and the strategies used to cope with them—can inform policy and practice by showing how such anxieties are constructed, interpreted, and embedded in lived experiences. As this methodological article does not present the full findings, these examples indicate potential applications rather than outcome-specific recommendations. In security policy, the findings may help identify anxieties related to military threats, psychosocial support, and public communication. In environmental policy, they may clarify how people understand threats such as pollution, climate change, and drought. Across both areas, the findings may also show which coping strategies respondents use. Such insights can support environmental education, risk communication, and locally adapted management measures.

## Limitations

Our approach has seven limitations. The first is the procedure's substantial time and coordination requirements. Developing a sufficiently shared interpretive approach required repeated training, iterative refinement of the codebook, and multiple online meetings among researchers. This process was particularly demanding because the coding team comprised nine researchers working across three countries and three languages. Moreover, because English was the primary language of communication and publication, aligning all three languages with it was sometimes challenging, and minor differences in meaning may have been lost. The procedure therefore required substantial preparation before coding could proceed in a stable and comparable manner. However, this limitation should be understood in relation to the complexity of cross-national qualitative teamwork. The Biographical-Narrative Toolkit for Anxieties and Coping Strategies developed during the study—especially the codebook, training materials, and paired-review procedure—now constitutes a transferable framework that can be adapted for future projects, reducing the need to build the entire process from scratch.

The second limitation relates to the conceptual status of anxiety in the empirical material. In the interviews, participants often used anxiety in a broad, everyday sense to refer not only to anxiety as defined in the study but also to related emotional states such as fear, stress, or anger. This created tension between the researchers' analytical framework and participants' narrative categorizations. This limitation also extends to translation. Although narrations could be translated without major losses of meaning, the exact lexical meaning of the translations of "anxiety" may not align perfectly across the target languages. We addressed this issue by emphasizing the study's conceptual understanding of anxiety during coder training, discussing doubtful passages within the team, and providing participants with our definition of the terminology. Nevertheless, the issue could not be fully eliminated. This reflects an inherent feature of biographical interviewing, in which participants retain control over the form and meaning of their views. The material therefore required continuous interpretive caution to maintain analytical focus without unduly constraining participants' self-descriptions.

The third limitation concerns the composition of the Ukrainian sample. Although the sample did not include people living under Russian occupation, participants represented a conflict-affected population because the interviews were conducted in person in Kyiv Oblast during the ongoing war, under conditions shaped by missile attacks, air-raid alerts, military mobilization, and persistent security uncertainty. Including participants from occupied territories would have created unacceptable risks for participants and researchers and prevented the study from being conducted safely and ethically. Consequently, the toolkit's transferability to populations living under occupation requires separate assessment under research conditions that adequately protect everyone involved.

The fourth limitation concerns the age gap between participants in the pilot cognitive interviews and those in the final study. The oldest participant in the pilot study was 50 years old, whereas the final sample included several participants in their 70 s and 80 s. Consequently, we cannot be certain that the pilot adequately addressed the cognitive and narrative challenges associated with older participants. Future studies should address this limitation by conducting pilot studies with participants who reflect the minimum and maximum ages of the planned final sample.

The fifth limitation concerns the nature of biographical interviews and their effect on the quality of the collected data. Retrospective biographical interviews require participants to reflect on their lives, which can be affected by memory biases such as omitting crucial information; forgetting events, dates, and numbers; and overestimating or underestimating the scale of experiences [24]. Furthermore, reconsidering life events retrospectively creates a risk of ad hoc reinterpretations that can substantially reshape participants' perceptions of their experiences and subsequent reflections. Emotions and memory can also reshape how past experiences are perceived and understood, potentially resulting in an imbalanced life-course account.

The sixth limitation concerns the assessment of coding consistency and reliability. Although the coders received common training and used the same coding materials, and their training outputs were reviewed comparatively by the principal investigator and the coordinator of biographical research, no formal quantitative measure of agreement was calculated. Instead, consistency was assessed qualitatively through detailed comparisons of coded excerpts, identification of discrepancies, and analysis of coders' questions and reports. This procedure helped refine the code tree, clarify instructions, and identify areas requiring further discussion. However, it did not yield a standardized reliability coefficient and therefore did not permit coder agreement to be expressed statistically. The reported consistency should thus be treated as a qualitative expert assessment rather than a formal measure of intercoder reliability. In addition, four of the nine coders also conducted interviews, creating a risk of confirmation bias during coding. Although this arrangement substantially reduced the number of researchers needed for the study procedure, overfamiliarity with the data may have influenced the analysis and should be considered when interpreting the results.

The seventh limitation concerns the availability of the qualitative data. Due to the biographical and highly contextual nature of the interviews and the sensitivity of the anxiety-related experiences discussed by participants, publishing complete transcripts or extended excerpts could create a risk of deductive disclosure [25,26] and cause potential harm or discomfort, even after the removal of direct identifiers. To protect participants’ confidentiality, full interviews are therefore not made publicly available. However, the data may be shared with individual researchers following a written agreement specifying the purpose and conditions of use, without permission to redistribute the transcripts or publish complete interviews or excerpts extending beyond a single coded segment.

## Related research article

None.

## For a published article

None.

## Declaration of generative AI and AI-assisted technologies in the manuscript preparation process

During the preparation of this work, the authors used NotebookLM [27] to refine the graphical abstract. After using this tool, the authors reviewed and edited the content as needed and take full responsibility for the content of the published article.

During the preparation of this work, the authors used ChatGPT [28] for improving the language quality of the manuscript. After using this tool, the authors reviewed and edited the text as needed and take full responsibility for the content of the published article.

## Ethics statements

All procedures involving human participants were conducted in accordance with applicable legal regulations, relevant ethical guidelines, and requirements for research involving human subjects. The study was approved by the Research Ethics Team of the Faculty of Sociology at Adam Mickiewicz University in Poznań, Poland, on April 24, 2024 (approval reference: ANSWER–24–04–2024). Participants' privacy and confidentiality were protected throughout the research process. All interview data were anonymized, and neither the manuscript nor any associated materials contain information that could identify individual participants. All participants provided informed consent before the interviews. They were informed about the study's purpose, the voluntary nature of participation, the incentives to which they were entitled, and their right to withdraw in accordance with the approved research protocol.

Because biographical interviews about anxiety and coping strategies can be emotionally challenging, we implemented trauma-informed procedures designed to minimize distress, preserve participants’ control over the interview, and protect the well-being of both participants and interviewers. Before data collection, the interviewers attended a meeting with a psychologist covering professional boundaries, recognition of emotional reactions, appropriate responses to severe distress, and available support. Participants could pause or withdraw from the study at any time without consequences. Interviewers monitored participants’ emotional state, confirmed whether they wished to continue if distress arose, and could end the interview when continuation appeared too burdensome. Participants’ well-being was checked after the interview, and the conversation was shifted to a neutral topic before its conclusion. The interviewers also had a list of local centers offering free psychological support; the list was available to both participants and interviewers. In addition, the interviews were coordinated by a psychologist with experience in crisis intervention, and interviewers could participate in group and individual sessions to discuss their experiences.

## Funding

This research was funded by the National Science Center (Poland) under SONATA 17 project: "ANSWER - ANxieties and Social coping strategies Within the last 50 years in the context of Natural EnviRonment: A Comparative Case Study of Poland, the Ukraine, and Hungary." (no. UMO-2021/43/D/HS6/01702).

## CRediT authorship contribution statement

**Krzysztof Mączka:** Conceptualization, Methodology, Software, Validation, Formal analysis, Investigation, Resources, Data curation, Writing – original draft, Writing – review & editing, Visualization, Project administration, Supervision, Funding acquisition. **Klaudia Rodziejczak:** Conceptualization, Methodology, Validation, Formal analysis, Investigation, Writing – original draft, Writing – review & editing, Visualization, Project administration, Funding acquisition. **Eszter Markó:** Methodology, Validation, Investigation, Writing – review & editing. **Ołena Bodnar-Potopnyk:** Methodology, Validation, Investigation, Writing – review & editing. **Oksana Khomiak:** Methodology, Validation, Investigation, Writing – review & editing. **Emőke Gondos:** Methodology, Validation, Investigation, Writing – review & editing. **Júlia Váradi:** Methodology, Validation, Investigation, Writing – review & editing. **Zuzanna Kurowska:** Methodology, Validation, Investigation, Writing – review & editing. **Barbara Sikora:** Methodology, Validation, Investigation, Writing – review & editing. **Maciej Milewicz:** Methodology, Writing – review & editing, Funding acquisition.

## Declaration of competing interest

The authors declare that they have no known competing financial interests or personal relationships that could have appeared to influence the work reported in this paper.

## Data Availability

Data will be made available on request.

## Appendix Supplementary materials

Image, application 1

Image, application 2

Image, application 3

Image, application 4

Image, application 5

Image, video 6 dummy alt text
