# Supplementary material for: Tracing anxious lives: a biographical-narrative toolkit for anxieties and coping strategies in three central and eastern European countries
Source: MethodsX. 2026 Aug 1;17:104084. doi: 10.1016/j.mex.2026.104084 (PMC13495090; doi:10.1016/j.mex.2026.104084)
Supplement: Supplementary file 1 [file mmc1.docx]

Cognitive Interview Scenario for Piloting Biographical Interviews

**General Instruction for the Researcher (not to be conveyed to the respondent):**

**The cognitive interview will be based on three techniques:**

1. Thinking aloud – the respondent should speak about their feelings while completing the questionnaire. We are interested in both 1) their observations, doubts regarding the content and form of individual questions, 2) and how they respond to assess how the tool works (e.g., whether they stay on topic or become verbose and open in their responses to individual questions). If the respondent forgets to "think aloud" at any point, they should be reminded to do so.
2. Probing questions – these are questions about the respondent's perception of the questions included in the interview or about explaining the respondent's behaviors, such as taking longer to think about a particular question. Probing questions will be asked after each section. When asking probing questions, several things should be kept in mind to maintain balance and neutrality:

a. We do not indicate our assessment of the respondent's emotional states, e.g., "You seemed embarrassed when answering this question, why?" We pay attention to facts, e.g., "You took a long time to answer this question, what was the reason?"

b. Instead of asking, for example, "Was question X difficult?" or "Was question X easy?" we ask, for instance, "Was this question easy or difficult?" or "How do you evaluate this question?"

c. Whenever there are doubts, we inquire "why?"

3. Observation – the researcher should note any non-standard behaviors of the respondent, if possible specifying precisely at which question they occurred. An observation protocol at the end of this file should be used for this purpose.

After the interview, the researcher should make notes listing the main problems that arose and suggestions for improvements. These notes should help in preparing a consolidated report from 3 interviews.

Before starting the actual interview, the participant should be informed that the main purpose of the meeting is to evaluate the tool for conducting biographical interviews. Their responses will not be analyzed or published. Detailed instructions for the study are included in the interview scenario. Below is just additional clarification for the cognitive interview section, which should be presented to the participant before starting the actual interview.

**Introduction for the Respondent (to be conveyed by the researcher):**

Thank you for agreeing to participate in the interview. As you know, the main goal of the research is to gather information about anxieties and coping strategies in Poland. The study will also be conducted in two other countries: Ukraine and Hungary.

We are at the beginning of the research. The purpose of this meeting is to test the scenario of our interview to assess how understandable it is and which elements need improvement from the perspective of the respondents.

We want to check whether the instructions and questions in our interview are clear, easy to understand, and whether they encourage sharing responses. Your participation in this interview will help us understand how these questions work and, as a result, improve them.

Please remember that there are no correct or incorrect answers. Please feel free to say whatever comes to mind regarding these questions.

The interview will be recorded so that no important insights are missed during our conversation. Your statements are confidential and will be anonymized to prevent your identification. Do you agree to this?

**Dispositions for Cognitive Interview**

**Explanation of the task**

• I will have a conversation with you about your anxieties and coping strategies throughout your life. While answering the questions, please, in addition to providing the answer to the question, talk about your feelings regarding the instructions and questions I ask, i.e., "think aloud" (especially if something is unclear).

• If you forget to "think aloud" at any point, I will remind you.

• The interview is divided into several parts. After each part, I will ask you a few probing questions.

**Warm-up: "Thinking Aloud"**

• At the beginning, as a warm-up, let's practice the "thinking aloud" technique briefly. Let's say I was asked, "How many windows are in my house?"

• If I were to talk about it aloud, I would say: "There is one window in the kitchen. Another one in the small room. In the big room, there are basically two windows next to each other, so they look like one window, but they open independently, so I'll treat them separately. Next to them are also balcony doors. I'm not sure if I should also count the balcony doors as windows, but I will do so, etc."

• Now I would like to ask you the same question. Please think aloud, how many windows are in your house/apartment?

• Thank you, now we can proceed to our interview.

**Completing the interview by the respondent and probing questions**

***• Probing questions should be asked considering: 1) what the respondent said "thinking aloud" and 2) our observations.***

***• If there is a need to modify a question (the respondent has already partially mentioned it) or to remove a question (the respondent has already answered it), the researcher can do so.***

**INSTRUCTION**

***We read the instruction to the respondent. After each paragraph, we ask for feedback.***

**Probing questions:**

1. How do you assess the clarity of this paragraph?
2. *If it is not clear*: What can be improved?
3. Other probing questions if necessary

**GENERAL ANXIETIES**

• Introduction to the first part of the study – probing questions

- How do you assess the clarity of the instructions?
- How do you assess the clarity of using the timeline?
- Other probing questions if necessary

**• Definitions**

- How do you assess the clarity of the anxiety definition?
- How do you assess the clarity of the coping definition?
- What anxiety means for you?
- What coping strategy means for you?
- Other probing questions if necessary

**• After each question included in the biographical interview scenario**

***Note: We aim for a natural flow of the interview, so if not all probing questions are used during the main response, at the end of the "general anxieties" section, we can present the unused probing questions for assessment.***

o How do you assess the clarity of this question?

o In the end: Was any question unnecessary in your opinion?

o Other probing questions if necessary

**ENVIRONMENTAL ANXIETIES**

• **Introduction to the second part of the study – probing questions:**

- How do you assess the clarity of the instructions?
- How do you assess the clarity of using the timeline?
- Other probing questions if necessary

**• After each question included in the biographical interview scenario**

***Note: We aim for a natural flow of the interview, so if not all probing questions are used during the main response, at the end of the "environmental anxieties" section, we can present the unused probing questions for assessment.***

- How do you assess the clarity of this question?
- In the end: Was any question unnecessary in your opinion?
- Other probing questions if necessary

**Comparative Part**

- **Introduction to the Third Part of the Study – Probing Questions:**
  - How do you asses the clarity of the instructions?
  - Other probing questions as needed
- **After each question in the biographical interview scenario:**

***Note: We aim for a natural flow of the interview, so if all probing questions are not used during the main response, we can present the unused probing questions for assessment at the end of the "general anxieties" section.***

- How do you assess the clarity of this question?
- Finally: Were any questions unnecessary in your opinion?
- Other probing questions as needed

**Post-interview calming procedure**

• How do you asses the conclusion of the interview?

• Are there any additional pieces of information that we should include in the interview conclusion? What are they?

• Were any questions or pieces of information unnecessary, according to the participant?

**Conclusion (after the whole bio scenario)**

1. How do you generally evaluate the interview?

2. What was easy for you during the question-answering process?

3. What was difficult for you during the question-answering process?

4. Were there any moments/questions during which you felt worse? Why? How could this be changed?

**Observation Protocol**

**Respondent Code (gender and age, e.g., M27):** ………………

In the cell, mark an X for the respective section and, if possible, enter the question/scenario element to which respondent reacted.

**Note: The protocol is not valuable in itself; it should support the researcher in asking probe questions.**

| Question | **Laugh** | **Head shaking** | **Changing answers multiple times** | **Prolonged thinking time** | **Others** |
| --- | --- | --- | --- | --- | --- |
| **e.g.,** |  |  |  |  |  |
|  |  |  |  |  |  |
|  |  |  |  |  |  |
|  |  |  |  |  |  |
|  |  |  |  |  |  |
|  |  |  |  |  |  |
|  |  |  |  |  |  |
|  |  |  |  |  |  |
|  |  |  |  |  |  |
