# Supplementary material for: Tracing anxious lives: a biographical-narrative toolkit for anxieties and coping strategies in three central and eastern European countries
Source: MethodsX. 2026 Aug 1;17:104084. doi: 10.1016/j.mex.2026.104084 (PMC13495090; doi:10.1016/j.mex.2026.104084)
Supplement: Supplementary file 2 [file mmc2.docx]

**Anxieties and coping strategies.**

Biographical interview tool

[Country] pilot study

Date: dd.mm.yyyy.

Content

[1.	Respondents	3](#_Toc162004652)

[2.	General notes about conducted interviews and level of engagement	4](#_Toc162004653)

[3.	Detailed notes about questions analyzed	5](#_Toc162004654)

1. Respondents

**3 biographical interviews were conducted.**

Respondents characteristics are described in the table below:

| Place of residence | Sex | Age | Education | Interview length |
| --- | --- | --- | --- | --- |
|  |  |  |  |  |
|  |  |  |  |  |
|  |  |  |  |  |
|  |  |  |  |  |
|  |  |  |  |  |
|  |  |  |  |  |
|  |  |  |  |  |
|  |  |  |  |  |
|  |  |  |  |  |
|  |  |  |  |  |

2. General notes about conducted interviews and level of engagement

0,5-1 page, in bullet points

3. Detailed notes about questions analyzed

| **Question** | **Content of the question (whole or part of the question)** | **Interview conclusions** | **Recommendation** |
| --- | --- | --- | --- |
| **Introduction** |  |  |  |
| **GENERAL ANXIETIES – INSTRUCTION** |  |  |  |
| **GENERAL ANXIETIES – QUESTIONS** |  |  |  |
| **ENVIRONMENTAL ANXIETIES – INSTRUCTION** |  |  |  |
| **ENVIRONMENTAL ANXIETIES – QUESTIONS** |  |  |  |
| **COMPARISION – INSTRUCTION** |  |  |  |
| **COMPARISION – QUESTIONS** |  |  |  |
| **POST-INTERVIEW CALMING PROCEDURE** |  |  |  |
