# Supplementary material for: Tracing anxious lives: a biographical-narrative toolkit for anxieties and coping strategies in three central and eastern European countries
Source: MethodsX. 2026 Aug 1;17:104084. doi: 10.1016/j.mex.2026.104084 (PMC13495090; doi:10.1016/j.mex.2026.104084)
Supplement: Supplementary file 3 [file mmc3.docx]

**Biographical interview scenario - ANSWER project**

**Screening interview:**

**Duration:** Approximately 15 minutes

Goals:

- To determine if the respondent is willing to participate in a longer biographical interview.
- To establish the general level of comfort of the respondent in discussing personal topics.

**Conversation:**

Introduction: Good morning/afternoon, my name is [Your Name], and I'm calling from [Organization Name]. I'm calling to confirm your participation in the study and to schedule a meeting. I would also like to ask a few questions and describe how the study will proceed. Our conversation should take no more than 15 minutes. Is this a good time for you?

**Description of the study:** First, let me briefly explain how our study will proceed. The study is being conducted at the [institution]. The results of this study will allow us to examine how different individuals cope with anxiety and compare this information between Poland, Ukraine, and Hungary. The technique we will be using for our conversation is called biographical interviewing. I would like to hear about the experiences (related to the world around us) throughout your life that have caused anxiety and how you have coped with it. Therefore, during our conversation, I will ask you to share your experiences and narrate them as if you were telling the story and being the narrator of your biography. The study will be divided into three main parts, during each of which I will ask questions about your experiences. Some of our conversations are shorter, others longer, so please book 3-4 hours, although our interview probably won't last that long. The study will be recorded by me so that nothing important is missed during our conversation. The recording will only be listened to for transcription purposes and will not be published anywhere. Your statements will be anonymized, which means that the author will not be identifiable, only age and gender will be provided. And if there are any personal information such as city names, names, or place names in the content, they will also be anonymized by us. Do you agree to be recorded during the study?

The meeting will take place at a mutually agreed location - it can be at your home or outside - for example, at the university or in a café. As a token of appreciation for participating in the study, we offer 250 PLN in vouchers. Do you have any questions so far?

**Checking readiness to discuss difficult topics**: As I mentioned, the conversation will be about anxieties and ways of coping with them throughout life. We understand that discussing such topics can be difficult, so I wanted to ask if you are ready to share such personal stories with the researcher and talk about your life? If yes -> establish the location and date of the interview, please note that it would be preferable for the interview to not be after a long exhausting day at work.

**Biographical Interview**

**Introduction (15 min.)**

1. **Greetings, Relationship and Comfort Building**

**Researcher**: Good morning/afternoon, my name is [Your Name], and I am a doctoral student/researcher at [University Name], at the [Department Name]. Before we begin our conversation, I would like to ask/add...

*This part of the interview is modified depending on our previous contact with the participant (e.g., upon entering the home or when scheduling the meeting). We can ask:*

- How are you feeling today?
- How has your day been so far?
- Address a topic related to the current situation, such as the location of the interview, travel, etc.

2. **Introduction and Obtaining Consent:**

**Researcher**: As you know, we have met to discuss your experiences related to anxiety and the coping mechanisms you employ. When speaking of anxieties, I mean that life is full of various worries; these worries sometimes weaken, sometimes intensify; our study concerns different anxieties that may have accompanied you throughout your life and are related to the world we live in.

The study will be recorded by me so that nothing important is missed during our conversation. The recording will only be listened to for transcription purposes for analysis and will not be published anywhere. Your statements will be anonymized, which means that the author will not be identifiable, only age and gender will be provided. And if there are any personal information such as city names, names, or place names in the content, they will also be anonymized by us. More detailed information is available in the information card, along with contact information for the project coordinator. Do you agree to be recorded? (*If yes - start recording and ask for consent to be recorded).*

The results of this study will allow us to examine how different individuals cope with anxiety and compare this information between Poland, Ukraine, and Hungary.

The study will be divided into three main parts, during each of which I will ask questions about your experiences.

The technique we will be using for our conversation is called biographical interview. I would like to hear about the experiences (related to the world around us) throughout your life that have caused anxiety and how you have coped with it. Therefore, during our conversation, I will ask you to share your experiences and narrate them as if you were telling the story and being the narrator of your biography.

During the first and second parts of the conversation, I will mainly listen to your story, occasionally taking notes. If necessary, I will ask questions to delve deeper into the topic.

The entire process should take no more than 3-4 hours.

During our conversation, we will also take short breaks. I plan to have a short break after Part I and Part II of the study, but if you need a moment to catch your breath during the interview, please let me know. (*We schedule breaks depending on the length of the interview; it's best to suggest a break at natural points, e.g., after the completion of Part I or Part II of the scenario, or when we see clear fatigue in the participant, etc. Even if the participant doesn't want any breaks at all, we'll still take at least one short break after Part II*).

Participation in the study is voluntary, and you may withdraw from the study at any stage.

Do you accept this form of conversation? Do you have any questions?

**General Anxiety (45-90 minutes)**

**Researcher: We will now move on to the first part of our study. To facilitate our conversation, we will use a timeline on which I will note your anxieties and coping mechanisms throughout your life. I will briefly write down what I hear on sticky notes and place them on the timeline after consulting with you*. As I mentioned earlier, I will ask you to narrate your story as if you were the narrator of your biography. However, it's up to you whether you start narrating chronologically or choose a different order.**

**Before we begin, I would like to read to you what we understand as "anxiety" and "coping" in our study to ensure that we are on the same page.**

**Definition of anxiety: Anxiety means feeling worse because you anticipate that something bad might happen that you won't be able to cope with.**

**Definition of coping: "Coping" means actions or ways of thinking used in a situation when you experience anxiety, aiming to improve well-being and reduce feelings of anxiety.**

*The researcher asks the following questions, and together with the participant, marks the respondent's answers and* ***approximate time (years of life and year)*** *on the timeline. *Instructions for using the timeline are in the notes at the end.*

1. Throughout their lives, people have different anxieties – some change, while others accompany us throughout our lives. Could you please talk about the most significant anxieties in your life? (*if necessary, you can add: You can take as long as you need to think about your answer*).
2. Please try to remember how you coped with these anxieties?
3. Could you think if there was a moment in your life when you started to look at this anxiety differently? Why? What happened? (*ask about specific anxieties if the participant hasn't mentioned them earlier*)

*If the participant moves on to discuss other anxieties and coping mechanisms, we follow along. If they find it difficult, we encourage them to share more memories and stories from their life. If necessary, we remind them that we are referring to "everyday anxieties related to the world around us." If the participant deviates too much from the topic of conversation, gently redirect them back on track. Repeat questions until the topic is done. If the researcher notices that the participant is discussing a situation that evokes emotions other than anxiety, ask supporting questions 9 or 10 and redirect the conversation to the appropriate topics.*

Supporting Questions:

1. Please recall a situation from last month that caused anxiety in you. What was it? (*ask if the participant struggles to start the story, then return to discussing the most significant anxieties in their life)*
2. Is this anxiety still present in your life?
3. When did you first notice this anxiety?
4. When did you first notice other anxieties? What were they?
5. Please try to remember how you coped with them?
6. Where did this coping mechanism come from?
7. Do you think anyone in your surroundings experiences similar anxieties? Who?
8. Could you recall and tell us about an event related to this anxiety/coping mechanism? (i*n case the participant only lists and doesn't narrate anything. Note: avoid pressuring the participant if any anxiety is emotionally difficult for them*).
9. What emotions does the situation/phenomenon evoke in you?
10. I wonder if the situation you described evokes anxiety in you? Or maybe it's a different emotion? What is it?

**Perception of Environmental Issues (30-60 minutes)**

*The researcher asks the following questions and collaborates with the participant to prepare a timeline, marking the respondent's answers and approximate time (years).*

**Researcher: Thank you for sharing those stories. Now, I would like to:**

A. **Deepen the topic that has already arisen in our conversation.**
B. **Address a new topic that has not yet been discussed in our conversation.**

*Option A requires flexibility. If the participant has talked a lot about environmental anxieties, we skip the second part and focus on other anxieties they have experienced in their life. If necessary, in this part, we can also delve deeper into environmental anxieties if the participant has not given them enough attention.*

**Researcher: I would like you to, similar to the previous part, talk about your anxieties and coping mechanisms throughout your life, but this time related to the natural environment. When talking about environmental anxieties, I mean that some people experience anxiety related to various natural phenomena, elements of nature, or changes occurring in the natural environment around us. Just like in the previous part, it's up to you whether you start narrating your story chronologically or choose a different order. To facilitate our conversation, we will again use a timeline on which we will note your anxieties and coping mechanisms.**

*If necessary, we can recall the definitions of anxiety and coping:*

**Definition of anxiety: Anxiety means feeling worse because you anticipate that something bad might happen that you won't be able to cope with.**

**Definition of coping: "Coping" means actions or thought processes used in a situation when you experience anxiety, aiming to improve well-being and reduce feelings of anxiety.**

1. As we have already discussed, people have different anxieties throughout their lives, sometimes also related to the natural environment. Could you tell me if you have experienced or are experiencing such anxieties?
2. If yes: Could you talk about the most significant anxieties related to the natural environment in your life?
3. How did you cope with these emotions?

*It may happen that the person does not have such anxieties. In that case, ask supporting questions 1-3. If the participant still does not mention any anxieties or if environmental problems evoke emotions other than anxiety (e.g., anger, sadness), leave the topic and move on to stage 3. If the researcher notices that the participant is discussing a situation that evokes emotions other than anxiety, ask supporting questions 7 or 8 and redirect the conversation to the appropriate topics.*

**Supporting Questions:**

1. Are environmental issues important to you in life? (*remember that some participants may not believe in climate change; in case of a negative reaction, it should be noted that we as researchers remain neutral on this topic and only want to understand the participant's opinion).*
2. What feelings do environmental issues evoke in you?
3. Are there any natural phenomena (or elements of the surrounding nature) that trigger anxiety in you?
4. When did you first feel anxiety related to the natural environment? What was it about?
5. When did you first notice other anxieties related to the environment? What were they?
6. Was there a moment in your life when your perception of environmental issues changed? What caused it? Did your feelings change in any way?
7. What emotions does the situation/phenomenon evoke in you?
8. I wonder if the situation you described evokes anxiety in you? Or maybe it's a different emotion? What is it?

**Comparison of the Natural Environment and Other Life Aspects (30 minutes)**

**Researcher: Thank you. Let's now look at both timelines/this timeline. They present your anxieties and coping mechanisms at different points in your life. Considering your life experiences and who you are today, please indicate which anxieties have had the greatest impact on your life? (if too difficult: which ones were/are the most important?). Please indicate a maximum of 5 anxieties.**

*It can be done silently, and the respondent marks them with colors. Then we ask the following questions:*

1. Why did you choose these anxieties?
2. Do you feel greater anxiety about environmental issues or other difficulties you have mentioned? Why? *(if the respondent did not have environmental anxieties, we skip this question)*

**Researcher: Let's now move on to coping mechanisms. Considering your life experiences and who you are today, please indicate which coping mechanisms for anxiety have had the greatest impact on your life? (*if too difficult: which ones were/are the most important?*). Please indicate a maximum of 5 coping mechanisms.**

*It can be done silently, and the respondent marks them with colors. Then we ask the following questions:*

1. Why are these coping mechanisms most important to you?
2. Are all these strategies effective for you?

**The end of the Interview (15 minutes)**

**Researcher: We will now be heading towards the end of our conversation. Therefore, I wanted to ask if there's anything else you'd like to add? ... We've talked a lot today about various anxieties, but such an interview is also an autobiographical experience; how have you experienced our conversation today (*or, if too difficult: what are your thoughts on this experience*)?**

**Thank you for your answers. I will now stop the recording.**

**Post-recording Questions (optional, if not addressed earlier):**

1. How was the conversation?
2. How do you feel after this conversation?
3. Did you take anything for yourself from this conversation?
4. Did anything surprise you during the conversation?
5. Is there anything we could improve in future interviews?

**Next Steps:**

- Thank you for your honest answers, assuring that everything was interesting and valuable.
- Informing about further research steps. (Transcription, interview analysis, writing scientific and popular science articles; you can provide a link to the project's Facebook page at this point)
- Handing out the voucher and gifts from UAM.
- Shifting the focus to another, fairly neutral topic (e.g., weather, travel to the interview location, pets, upcoming holidays).
- Farewell.

**Additional Tips and Remarks:**

1. **Individual experiences:** Encourage sharing personal stories and experiences.
2. **Flexibility and adjustment:** Remain flexible in adapting the script during the interview to better respond to the respondent's needs and experiences. Remember to adjust the language to the interviewee - it is recommended to simplify questions if necessary. Tip: Ask the question in a way that your grandparent would understand.
3. **Building relationship and comfort** starts already during the first contact, such as a preliminary interview over the phone.
4. Keep in mind that the interview may be exhausting for the interviewee due to its duration (the longest pilot lasted 3 hours and 40 minutes). When scheduling, mention this and try to set a time when the person is not too tired, for example, after a long day at work.
5. **Bring 2x A3 paper, markers, pens, sticky notes (preferably small size, larger ones can be cut in half), a recorder, tissues** (the interviewee may cry).
6. **The conversation involves emotionally difficult topics.** Do not judge the interviewee, their anxieties, or the effectiveness of coping mechanisms. Do not give advice on how to deal with the anxieties discussed, even after the interview ends. Adopt an empathetic but neutral attitude.
7. Do not engage in debates with interviewees, especially on political topics.
8. If the interview would be too overwhelming for the interviewee, assure them that we can stop the conversation at any time, and if necessary, proceed to terminate the interview, hand over the voucher, and refer to point 9.
9. **If the interviewee feels mentally unwell after the conversation, redirect them to the nearest support center from the prepared list of free psychological assistance centers and helplines.**
10. **TIMELINE.**
    a. To conduct the study, we need: an A3 paper (or larger format but remember that space on the interviewee's table may be limited), sticky notes, markers, and pens. We should have at least 4 colors of sticky notes (2 colors for general anxieties and environmental ones, 2 colors for coping strategies for general anxieties and environmental ones).
    b. Before the study, draw the timeline with a marker on a sheet of paper (a long black arrow, the phrase "Timeline of Your Life" at the top).
    c. During the study, as the interviewee tells their stories, write down anxieties and coping strategies on sticky notes, and then place them on the timeline. We can also mark important life events on the timeline (e.g., birth, starting/ending school, marriage, birth of children, death of loved ones, etc.). Additionally, key events related to anxieties can be marked, e.g., witnessing a car accident, illness, etc.
    d. Consult the interviewee on their content and placement. Flexibility - consultation should be done in a way that allows us to avoid interrupting the respondent's story.
    e. It may happen that the interviewee applies one coping strategy to more than one anxiety; this should be marked on the timeline.
    f. In the comparative part, the interviewee can mark anxieties and coping strategies themselves.
    g. In the case of online research, we use a program such as https://jamboard.google.com/d/1FtT5iZRUAUtMvU4tLl37OhUTOgCvUxpX5xxB0AtbNi4/edit?usp=sharing Then, the researcher both writes down anxieties and coping strategies and marks them in the comparative part (this eliminates the problem of translating how to do it by the interviewee and technical problems). This program also has "sticky notes" and a "pen" for marking. During the study, one or two timelines can be used, depending on how many anxieties are presented by the interviewee. If there are a few, one timeline will be more convenient.
    h. **REMEMBER - TAKE A PICTURE OF THE TIMELINE AT THE END OF THE INTERVIEW!!!!**
    i. If in part II of the study, the interviewees are surprised by the change of topic from general anxieties to environmental ones, we should re-explained the purpose of the study and explained that we did not mention it earlier to avoid suggesting answers related to the natural environment.
    j. Positive reflection - despite the interview being on a difficult topic, talking about coping strategies can enhance the interviewees' sense of agency and control.
