# Supplementary material for: Tracing anxious lives: a biographical-narrative toolkit for anxieties and coping strategies in three central and eastern European countries
Source: MethodsX. 2026 Aug 1;17:104084. doi: 10.1016/j.mex.2026.104084 (PMC13495090; doi:10.1016/j.mex.2026.104084)
Supplement: Supplementary file 4 [file mmc4.docx]

**Report from biographical interviews**

**Interviewer:**

**Interview code (same as recording name):**

**Location of the interview:**

**Report:**

1. **General impressions**

• Description of respondents' initial reactions to the research questions.

• Overall impression of respondents' comfort during the conversation (when they felt uncomfortable and when comfortable).

2. **The course of the interview**

• Brief description of interaction and conversation dynamics.

3. **Observed difficulties**

• Any challenges the researcher or respondent encounters during the interview (e.g., difficult questions, emotional moments).

4. **Reflection on the method**

• Evaluation of the effectiveness of the interview (scenario, methods) in achieving research goals.

• Any suggestions for modifying the scenario/method for the future if necessary.

5. **Organizational and technical issues**

• Any organizational problems encountered (e.g., scheduling, technical obstacles, interview interferences).
